# Cardiovascular Outcomes With Icosapent Ethyl by Baseline Low‐Density Lipoprotein Cholesterol: A Secondary Analysis of the REDUCE‐IT Randomized Trial

**DOI:** 10.1161/JAHA.124.038656

**Published:** 2025-02-19

**Authors:** Rahul Aggarwal, Deepak L. Bhatt, Ph. Gabriel Steg, Michael Miller, Eliot A. Brinton, Richard L. Dunbar, Steven B. Ketchum, Jean‐Claude Tardif, Fabrice M. A. C. Martens, Christie M. Ballantyne, Michael Szarek, R. Preston Mason, REDUCE‐IT Investigators

**Affiliations:** ^1^ Brigham and Women’s Hospital Heart and Vascular Center Harvard Medical School Boston MA; ^2^ Mount Sinai Fuster Heart Hospital, Icahn School of Medicine at Mount Sinai New York NY; ^3^ Université de Paris, FACT (French Alliance for Cardiovascular Trials), Assistance Publique–Hôpitaux de Paris, Hôpital Bichat, INSERM Unité 1148 Paris France; ^4^ Department of Medicine Crescenz Veterans Affairs Medical Center and University of Pennsylvania School of Medicine Philadelphia PA; ^5^ Utah Lipid Center Salt Lake City UT; ^6^ Amarin Pharma, Inc. Bridgewater NJ; ^7^ Department of Medicine Perelman School of Medicine at the University of Pennsylvania Philadelphia PA; ^8^ Montreal Heart Institute, Université de Montréal Montreal Quebec Canada; ^9^ Department of Cardiology Amsterdam UMC Amsterdam The Netherlands; ^10^ Department of Medicine Baylor College of Medicine, and the Texas Heart Institute Houston TX; ^11^ Division of Cardiology University of Colorado School of Medicine Aurora CO; ^12^ CPC Clinical Research Aurora CO; ^13^ State University of New York, Downstate Health Sciences University Brooklyn NY; ^14^ Elucida Research LLC Beverly MA

**Keywords:** cardiovascular outcomes, icosapent ethyl, low‐density lipoprotein cholesterol, Quality and Outcomes, Clinical Studies

## Abstract

**Background:**

The efficacy of icosapent ethyl among patients with very well‐controlled baseline low‐density lipoprotein cholesterol (LDL‐C) is unknown.

**Methods:**

In this post hoc analysis of the REDUCE‐IT (Reduction of Cardiovascular Events With Icosapent Ethyl–Intervention Trial) randomized clinical trial, statin‐treated patients with high cardiovascular risk, elevated triglycerides (135–499 mg/dL), and baseline LDL‐C of 41 to 100 mg/dL were included. Patients were randomized to icosapent ethyl (2 g twice daily) or placebo and then post hoc stratified by baseline LDL‐C (<55 mg/dL versus ≥55 mg/dL). The primary composite end point included cardiovascular death, nonfatal myocardial infarction, nonfatal stroke, coronary revascularization, or unstable angina.

**Results:**

Among 8175 patients with baseline LDL‐C data, 7117 (87.1%) had LDL‐C ≥55 mg/dL and 1058 (12.9%) had LDL‐C <55 mg/dL. In patients with LDL‐C <55 mg/dL, the rate of the primary composite end point was lower in the icosapent ethyl group (16.2% versus 22.8%) than in the placebo group (hazard ratio [HR], 0.66 [95% CI, 0.50–0.87]; absolute risk reduction, 6.6%; *P*=0.003). Among patients with LDL‐C ≥55 mg/dL, a primary composite end point event occurred in a lower proportion of patients in the icosapent ethyl group (17.4% versus 21.9%) than in the placebo group (HR, 0.76 [95% CI, 0.69–0.85]; absolute risk reduction, 4.5%; *P*<0.0001). No significant interaction was observed between baseline LDL‐C and treatment group (*P* for interaction=0.40). Findings were consistent among secondary cardiovascular end points and in sensitivity analyses.

**Conclusions:**

Among statin‐treated patients with elevated triglycerides and high cardiovascular risk, icosapent ethyl reduced the rate of cardiovascular end points irrespective of baseline LDL‐C, including among eligible patients with optimal LDL‐C control.

**Registration:**

URL: https://www.clinicaltrials.gov; Unique identifier: NCT01492361.

**Nonstandard Abbreviations and Acronyms**
EPA: eicosapentaenoic acid
EVAPORATE: Effect of Vascepa on Improving Coronary Atherosclerosis in People With High Triglycerides Taking Statin Therapy
JELIS: Japan EPA Lipid Intervention Study
PROMINENT: Pemafibrate to Reduce Cardiovascular Outcomes by Reducing Triglycerides in Patients With Diabetes
REDUCE‐IT: Reduction of Cardiovascular Events With Icosapent Ethyl–Intervention Trial
RESPECT‐EPA: Randomized Trial for Evaluation in Secondary Prevention Efficacy of Combination Therapy–Statin and Eicosapentaenoic Acid
STRENGTH: Long‐Term Outcomes Study to Assess Statin Residual Risk With Epanova in High Cardiovascular Risk Patients With Hypertriglyceridemia
TEAE: treatment‐emergent adverse event

**Clinical Perspective**

**What Is New?**

- In this post hoc analysis of REDUCE‐IT (Reduction of Cardiovascular Events With Icosapent Ethyl–Intervention Trial), 8175 statin‐treated patients with high cardiovascular risk and elevated triglycerides were randomized to icosapent ethyl or placebo and then stratified by baseline low‐density lipoprotein cholesterol control (<55 mg/dL versus ≥55 mg/dL).
- Icosapent ethyl significantly reduced the primary composite end point of cardiovascular events by 34% among patients with very well‐controlled low‐density lipoprotein cholesterol.
- Findings were consistent across the full range of baseline low‐density lipoprotein cholesterol levels and for a variety of cardiovascular outcomes.

**What Are the Clinical Implications?**

- Icosapent ethyl is effective in reducing cardiovascular events among indicated patients irrespective of baseline low‐density lipoprotein cholesterol.
- Icosapent ethyl should be considered for statin‐treated patients with elevated triglycerides and high cardiovascular risk even if low‐density lipoprotein cholesterol is optimally controlled.

Patients with mildly to moderately elevated triglycerides have an increased risk of cardiovascular disease.^1^, ^2^ Icosapent ethyl is a purified eicosapentaenoic acid (EPA) ethyl ester that has been shown to decrease triglyceride levels,^3^ in addition to favorable effects on plasma levels of oxidized low‐density lipoproteins, various plasma markers of inflammation, and cell membrane stabilization.^4^, ^5^, ^6^, ^7^, ^8^

The REDUCE‐IT (Reduction of Cardiovascular Events With Icosapent Ethyl–Intervention Trial) randomized clinical trial demonstrated that statin‐treated patients with low‐density lipoprotein cholesterol (LDL‐C) of ≤100 mg/dL, elevated triglycerides (135–499 mg/dL), and high cardiovascular risk experienced fewer cardiovascular end points with icosapent ethyl compared with placebo.^3^, ^9^, ^10^, ^11^, ^12^, ^13^, ^14^, ^15^ Recent evidence indicates benefit of intensive LDL‐C lowering, with guidelines recommending an LDL‐C goal of ≤55 mg/dL in especially high‐risk patients.^16^, ^17^ Whether the beneficial effects of icosapent ethyl are consistent across a range of baseline LDL‐C levels, especially among those with very well‐controlled LDL‐C, remains unknown. Because the triglyceride and cholesterol pathways have substantial overlap,^18^ evaluating treatment heterogeneity among drugs targeting these markers is important. Understanding if icosapent ethyl reduces cardiovascular events in patients with optimal LDL‐C on statin therapy can inform therapy decisions.

We performed a post hoc secondary analysis of REDUCE‐IT to determine the efficacy of icosapent ethyl compared with placebo for cardiovascular event reduction among patients stratified by baseline LDL‐C.

## Methods

### Study Design and Patient Population

Patients in REDUCE‐IT were included. REDUCE‐IT has been described previously.^3^, ^19^ In brief, REDUCE‐IT was a double‐blind, randomized, placebo‐controlled trial enrolling patients from 473 sites in 11 countries. Randomization occurred between November 28, 2011, and August 4, 2016. Patients were randomized to icosapent ethyl (2 g twice daily with food) or placebo. Patients aged ≥45 years with cardiovascular disease or ≥50 years with diabetes and ≥1 cardiovascular risk factor were included. Patients were further selected to be on statin treatment consistently for at least 4 weeks. Patients were included if they had triglycerides of 135 to 499 mg/dL and LDL‐C of 41 to 100 mg/dL. Patients were excluded if they had severe heart failure, acute severe liver disease, or a glycated hemoglobin >10.0%. Other key inclusion and exclusion criteria, as well as detailed methods and protocol, have been described previously.^3^ The data that support the findings and the methods used to conduct the study may be made available upon reasonable request to the corresponding author.

For this analysis, patients were stratified by baseline LDL‐C to <55 mg/dL or ≥55 mg/dL. This threshold was consistent with the LDL‐C target for patients with cardiovascular disease by the European Society of Cardiology guidelines.^16^ In sensitivity analyses (see Statistical Analysis section), patients were stratified by LDL‐C <70 mg/dL and ≥70 mg/dL, as this threshold was consistent with American College of Cardiology and American Heart Association guidelines for lipid control.^20^ These thresholds were selected to stratify patients to well‐controlled and less‐controlled LDL‐C groups. Baseline LDL‐C was determined as the last nonmissing measurement of LDL‐C before randomization. LDL‐C was obtained via preparative ultracentrifugation. If not available by preparative ultracentrifugation, then another LDL‐C value before randomization was obtained, preferably by direct measurement of LDL‐C, and if not available, then by Friedewald calculation (for patients with triglycerides <400 mg/dL) or otherwise by the Martin–Hopkins equation.^21^, ^22^

### Outcomes

The primary composite end point included cardiovascular death, nonfatal myocardial infarction (MI), nonfatal stroke, coronary revascularization, or unstable angina. Multiple secondary end points were studied: cardiovascular death, nonfatal MI, or nonfatal stroke (termed key secondary composite end point); cardiovascular death or nonfatal MI; fatal or nonfatal MI; urgent or emergent revascularization; cardiovascular death; hospitalization for unstable angina; fatal or nonfatal stroke; all‐cause death, nonfatal MI, nonfatal stroke; and all‐cause death. Safety end points included a composite of treatment‐emergent adverse events (TEAEs), including severe TEAEs and drug‐related TEAEs, as well as cause‐specific TEAE (ie, atrial fibrillation/atrial flutter).

### Statistical Analysis

After stratifying patients by baseline LDL‐C (<55 mg/dL or ≥55 mg/dL), end points were assessed by treatment group with intention‐to‐treat analyses for time to first event. Log‐rank *P* values were reported from Kaplan–Meier analysis, stratified by geographic region, cardiovascular risk category (established cardiovascular disease or diabetes plus risk factors), and use of ezetimibe. Hazard ratios (HRs) and 95% CIs were determined using a Cox proportional hazards model computed to determine the risk of the primary and key secondary end points according to the use of icosapent ethyl compared with placebo. To assess heterogeneity in treatment effects, interaction analyses were performed. In these analyses, differences by baseline LDL‐C group were examined with Cox proportional hazard models including an interaction term for baseline LDL‐C group and treatment group.

Heterogeneity in the relative treatment effect was assessed by evaluating continuous baseline LDL‐C with a Cox proportional hazard model incorporating treatment assignment, a natural cubic spline of baseline LDL‐C, and the interaction between treatment and the spline. Knots were specified at the 25th percentile, median, and 75th percentile of baseline LDL‐C, and a Wald test was used to assess the significance of the interaction term.

To ensure robustness of results, multiple sensitivity analyses were conducted. First, the primary composite end point was determined among patients with cardiovascular disease history. Second, the entire cohort was stratified by a less intensive LDL‐C threshold: LDL‐C <70 mg/dL and LDL‐C ≥70 mg/dL.

Four patients were excluded for missing baseline LDL‐C values (<0.05%). A *P* threshold of <0.05 was considered significant for the overall analyses, and a *P* threshold of <0.10 was considered significant for the interaction analyses, consistent with prior studies.^23^ Analyses were conducted with SAS 9.4 (SAS Institute Inc., Cary, NC).^24^ REDUCE‐IT was approved by the appropriate institutional review boards, public health authorities, and ethics committees. Informed consent was obtained from participants. REDUCE‐IT was registered on ClinicalTrials.Gov (NCT01492361).

## Results

REDUCE‐IT included 8179 patients. After excluding 4 patients with missing baseline LDL‐C data, the final study population consisted of 8175 patients. Of these patients, 7117 (87.1%) had baseline LDL‐C ≥55 mg/dL, and 1058 (12.9%) had LDL‐C <55 mg/dL.

In the LDL‐C ≥55 mg/dL group, median age was 64.0 (interquartile range [IQR], 57.0–69.0) years, 2091 were women (29.4%), and 5055 (71.0%) had a history of cardiovascular disease. Median triglycerides were 215.5 (IQR, 175.0–270.0) mg/dL, and median LDL‐C was 78.0 (IQR, 67.0–91.0) mg/dL. In the LDL‐C <55 mg/dL group, median age was 64.0 (IQR, 58.0–70.0) years, 265 (25.0%) were women, and 726 (68.6%) had a history of cardiovascular disease. Median triglycerides were 228.0 (IQR, 182.0–300.5) mg/dL, and median LDL‐C was 47.5 (IQR, 42.0, 52.0) mg/dL. Baseline characteristics by LDL‐C group are shown in Table 1 and by treatment group in Table S1.

**Table 1:** Baseline Characteristics by LDL‐C Groups

	Overall*		
	Baseline LDL‐C ≥55 mg/dL (N=7117)	Baseline LDL‐C <55 mg/dL (N=1058)	*P* value†
Age, y, median (quartiles 1–3)	64.0 (57.0–69.0)	64.0 (58.0–70.0)	0.007
Age ≥65 y, n (%)‡	3241 (45.5)	521 (49.2)	0.02
Female, n (%)	2091 (29.4)	265 (25.0)	0.004
Hispanic or Latino ethnicity, n (%)	313 (4.4)	32 (3.0)	0.04
Race, n (%)§			<0.0001
White	6452 (90.7)	924 (87.3)	
Black or African American	137 (1.9)	21 (2.0)	
Asian	350 (4.9)	95 (9.0)	
Other or multiple	178 (2.5)	17 (1.6)	
United States, n (%)	2704 (38.0)	438 (41.4)	0.03
BMI, kg/m^2^, median (quartiles 1–3)	30.8 (27.8–34.6)	30.9 (28.1–34.4)	0.93
BMI ≥30 kg/m^2^, n (%)‡	4086 (57.4)	606 (57.3)	0.96
Stratification factors, n (%)			
Location			<0.0001
Westernized	5038 (70.8)	769 (72.7)	
Eastern Europe	1885 (26.5)	221 (20.9)	
Asia Pacific	194 (2.7)	68 (6.4)	
Cardiovascular risk category—as randomized			0.11
Cardiovascular risk category 1 (secondary prevention)	5055 (71.0)	726 (68.6)	
Cardiovascular risk category 2 (primary prevention)	2062 (29.0)	332 (31.4)	
Ezetimibe use	451 (6.3)	73 (6.9)	0.49
Statin intensity and diabetes status, n (%)			
Statin intensity			0.04
Low	455 (6.4)	66 (6.2)	
Moderate	4482 (63.0)	624 (59.0)	
High	2155 (30.3)	359 (33.9)	
Missing	25 (0.4)	9 (0.9)	
Diabetes			<0.0001
Type 1	48 (0.7)	9 (0.9)	
Type 2	4042 (56.8)	684 (64.7)	
No diabetes at baseline	3026 (42.5)	363 (34.3)	
Missing	1 (0.0)	2 (0.2)	
Laboratory measurements			
Creatinine clearance >30 and <60 mL/min, n (%)	771 (10.8)	119 (11.2)	0.66
hs‐CRP (mg/L), median (quartiles1–3)	2.2 (1.1–4.5)	2.2 (1.0–4.4)	0.50
Triglycerides (mg/dL), median (quartiles 1–3)	215.5 (175.0–270.0)	228.0 (182.0–300.5)	<0.0001
Triglyceride category, n (%)			0.006
<150 mg/dL	750 (10.5)	91 (8.6)	
150 to <200 mg/dL	2104 (29.6)	280 (26.5)	
≥200 mg/dL	4263 (59.9)	687 (64.9)	
Triglyceride tertiles, n (%)			<0.0001
Lowest (≥81.25 to ≤190 mg/dL)	2445 (34.4)	314 (29.7)	
Middle (>190 to ≤250 mg/dL)	2379 (33.4)	317 (30.0)	
Upper (>250 to ≤1401 mg/dL)	2293 (32.2)	427 (40.4)	
Triglycerides ≥200 mg/dL and HDL‐C ≤35 mg/dL, n (%)	1302 (18.3)	315 (29.8)	<0.0001
HDL‐C (mg/dL), median (quartiles 1–3)	40.5 (35.5–46.0)	37.5 (32.0–44.0)	<0.0001
Apo B (mg/dL), median (quartiles 1–3)	84.0 (75.0–95.0)	65.0 (57.0–73.0)	<0.0001
LDL‐C/apo B, median (quartiles 1–3)	0.9 (0.8–1.0)	0.7 (0.6–0.8)	<0.0001
Atherosclerosis index in plasma, median (quartiles 1–3)	0.4 (0.2–0.5)	0.4 (0.3–0.6)	<0.0001
LDL‐C (mg/dL), median (quartiles 1–3)	78.0 (67.0–91.0)	47.5 (42.0–52.0)	<0.0001
EPA (μg/mL), median (quartiles 1–3)	26.3 (17.3–40.2)	24.3 (16.1–38.2)	0.004
Medications taken at baseline, n (%)			
Antidiabetes	3738 (52.5)	644 (60.9)	<0.0001
Antihypertensive	6788 (95.4)	998 (94.3)	0.14
Antiplatelet‖	5659 (79.5)	830 (78.4)	0.42
1 antiplatelet	4242 (59.6)	580 (54.8)	0.003
≥2 antiplatelets	1417 (19.9)	250 (23.6)	0.005
Anticoagulant	665 (9.3)	110 (10.4)	0.28
Anticoagulant plus antiplatelet	229 (3.2)	45 (4.3)	0.08
No antithrombotic	1022 (14.4)	163 (15.4)	0.37
ACEi	3733 (52.5)	508 (48.0)	0.007
ARB	1878 (26.4)	325 (30.7)	0.003
ACEi or ARB	5520 (77.6)	817 (77.2)	0.81
β Blockers	5020 (70.5)	758 (71.6)	0.46
Statin	7092 (99.6)	1049 (99.1)	0.02

In general, the baseline value is defined as the last nonmissing measurement obtained before randomization. The baseline LDL‐C value obtained via preparative ultracentrifugation was used unless this value was missing. If missing, then another LDL‐C value was used, with prioritization of values obtained from LDL‐C direct measurements, followed by LDL‐C derived by the Friedewald calculation method (only for subjects with triglycerides <400 mg/dL), and finally LDL‐C derived using the calculation published by Johns Hopkins University investigators. For all other lipid and lipoprotein marker parameters, wherever possible, baseline was derived as the arithmetic mean of the visit 2 (day 0) value and the preceding visit 1 (or visit 1.1) value. If only 1 of these values was available, the single available value was used as baseline. Tertiles for triglycerides are based on the overall intention‐to‐treat population. ACEi indicates angiotensin‐converting enzyme inhibitor; apo B, apolipoprotein B; ARB, angiotensin receptor blocker; BMI, body mass index; EPA, eicosapentaenoic acid; HDL‐C, high‐density lipoprotein cholesterol; hs‐CRP, high‐sensitivity C‐reactive protein; and LDL‐C, low‐density lipoprotein cholesterol.

* Baseline LDL‐C is missing for 3 subjects in the icosapent ethyl arm and 1 subject in the placebo arm.

^†^ *P* values are reported from a χ^2^ test for categorical variables and Wilcoxon test for continuous variables. Missing categories are excluded from any comparisons.

^‡^ *P* value is based on <65 years and ≥65 years for age; and <25 kg/m^2^, ≥25 to <30 kg/m^2^ and ≥30 kg/m^2^ for BMI category.

^§^ *P* value is based on the race categories as listed herein. The category “other or multiple” also includes American Indian, Alaskan Native, Native Hawaiian, and Other Pacific Islander.

^‖^ Antiplatelet medications were classified as dual if both components have a regulatory approval affirming antiplatelet effects. Combinations in which 1 element lacks such regulatory approval were excluded (eg, aspirin+magnesium oxide is classified as a single agent because the latter component is not approved as an antiplatelet agent).

### Primary Composite End Point

In the overall REDUCE‐IT population, a primary composite end point event (cardiovascular death, nonfatal MI, nonfatal stroke, coronary revascularization, or unstable angina) occurred in 17.2% (705/4089) of patients in the icosapent ethyl group and in 22.0% (901/4090) of patients in the placebo group (HR, 0.75 [95% CI, 0.68–0.83]; absolute risk reduction [ARR], 4.8%; *P*<0.0001).

**Figure 1:**
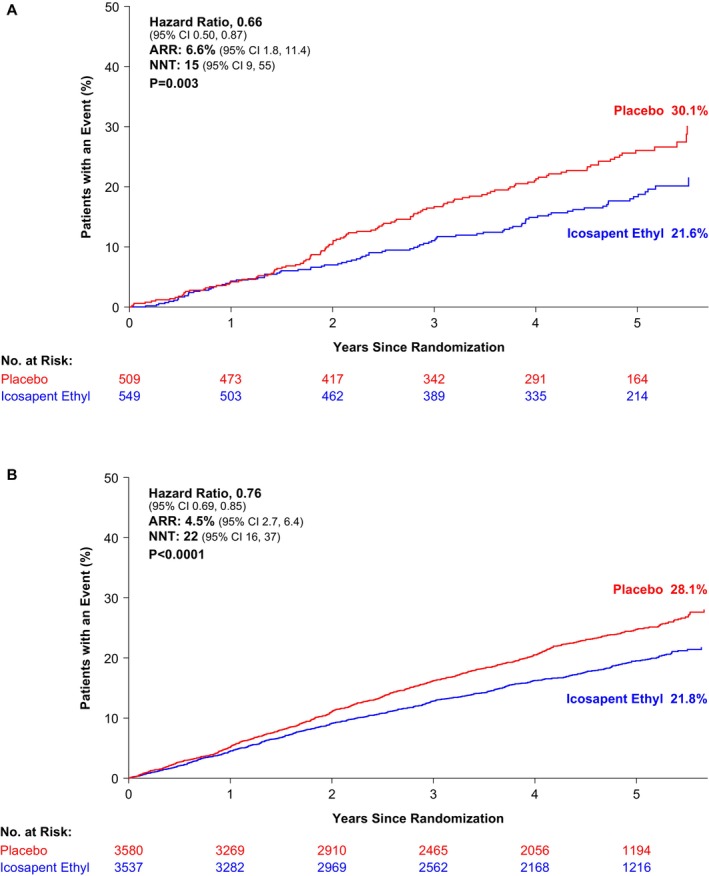
Kaplan–Meier plot of primary composite end point by baseline LDL‐C. Presented are the Kaplan–Meier plots of the primary end point (cardiovascular death, nonfatal myocardial infarction, nonfatal stroke, coronary revascularization, or unstable angina). Patients were randomized to icosapent ethyl (2 g twice daily) or placebo. Patients were stratified by baseline LDL‐C (<55 mg/dL and ≥55 mg/dL). Interaction *P* value for treatment effect by baseline LDL‐C group was 0.40. **A**, LDL‐C <55 mg/dL (N=1058). **B**, LDL‐C ≥55 mg/dL (N=7117). ARR indicates absolute risk reduction; LDL‐C, low‐density lipoprotein cholesterol; and NNT, number needed to treat.

Similar findings were observed in the 2 strata of baseline LDL‐C. Among patients with LDL‐C <55 mg/dL, a primary composite end point event occurred in 16.2% (89/549) of patients in the icosapent ethyl group and in 22.8% (116/509) of patients in the placebo group (HR, 0.66 [95% CI, 0.50–0.87]; ARR, 6.6%; number needed to treat, 15; *P*=0.003; Figure 1). In patients with LDL‐C ≥55 mg/dL, the rate of the primary composite end point was 17.4% (616/3537) in the patients of the icosapent ethyl group and 21.9% (785/3580) in those of the placebo group (HR, 0.76 [95% CI, 0.69–0.85]; ARR, 4.5%; number needed to treat, 22; *P*<0.0001; Figure 1). No significant interaction was observed between baseline LDL‐C and treatment group (*P* for interaction=0.40).

**Figure 2:**
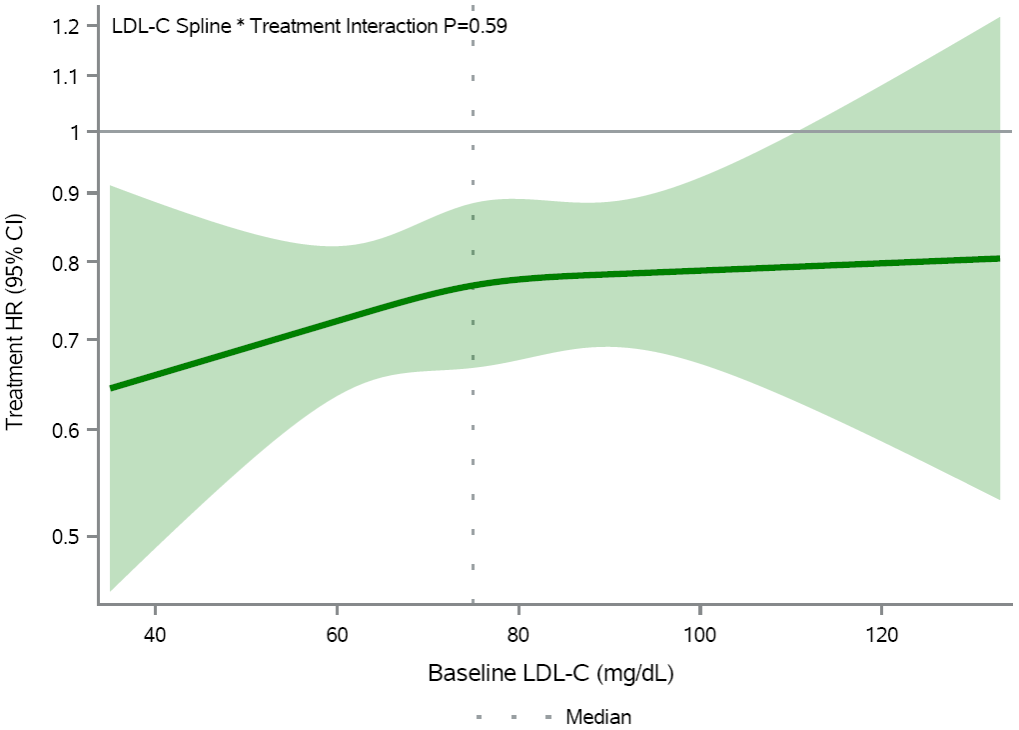
Efficacy of icosapent ethyl by baseline LDL‐C. Presented is the treatment hazard ratio of icosapent ethyl compared with placebo (*y* axis) for the primary composite end point by baseline LDL‐C (*x* axis). The primary composite end point included cardiovascular death, nonfatal myocardial infarction, nonfatal stroke, coronary revascularization, or unstable angina. Baseline LDL‐C was evaluated continuously in this analysis using a natural cubic spline with knots placed at the 25th percentile, median, and 75th percentile of LDL‐C. A hazard ratio of <1 indicates benefit with icosapent ethyl. The *x* axis range covers the 1st to 99th percentile of LDL‐C values (35–133 mg/dL). HR indicates hazard ratio; and LDL‐C, low‐density lipoprotein cholesterol.

In analyses evaluating baseline LDL‐C as a continuous variable, no difference in relative treatment effect by LDL‐C was observed (*P* for interaction=0.59; Figure 2), indicating consistent reductions in the primary outcome across patients with well‐managed LDL‐C.

### Secondary End Points

**Figure 3:**
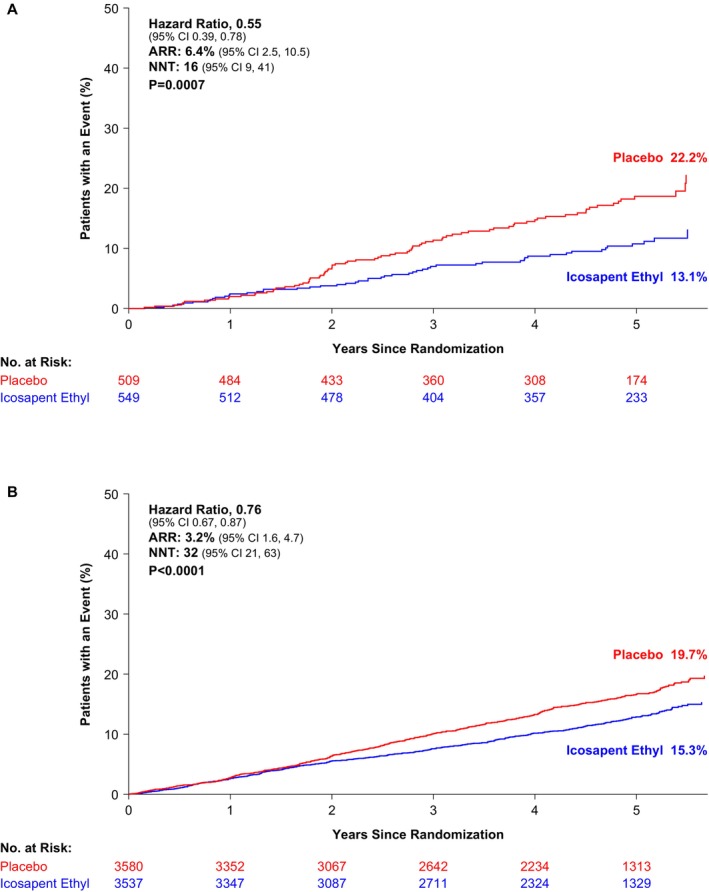
Kaplan–Meier plot of key secondary composite end point by baseline LDL‐C. Presented are the Kaplan–Meier plots of the key secondary composite end point (cardiovascular death, nonfatal myocardial infarction, or nonfatal stroke). Patients were randomized to icosapent ethyl (2 g twice daily) or placebo. Patients were stratified by baseline LDL‐C (<55 mg/dL and ≥55 mg/dL). Interaction *P* value for treatment effect by baseline LDL‐C group was 0.11. ARR indicates absolute risk reduction; LDL‐C, low‐density lipoprotein cholesterol; and NNT, number needed to treat. **A**, LDL‐C <55 mg/dL (N=1058). **B**, LDL‐C ≥55 mg/dL (N=7117).

The rate of the key secondary composite end point (cardiovascular death, nonfatal MI, or nonfatal stroke) in the overall population was 11.2% (459/4089 patients) in the icosapent ethyl group and 14.8% (606/4090 patients) in the placebo group (HR, 0.74 [95% CI, 0.65–0.83]; ARR, 3.6%; *P*<0.0001). Among patients with LDL‐C <55 mg/dL, a key secondary composite end point event occurred in 9.5% (52/549) of patients in the icosapent ethyl group and in 15.9% (81/509) of patients in the placebo group (HR, 0.55 [95% CI, 0.39–0.78]; ARR, 6.4%; number needed to treat, 16; *P*=0.0007; Figure 3). In patients with LDL‐C ≥55 mg/dL, the rate of the key secondary composite end point was 11.5% (407/3537 patients) in the icosapent ethyl group and 14.7% (525/3580 patients) in the placebo group (HR, 0.76 [95% CI, 0.67–0.87]; ARR, 3.2%; number needed to treat, 32; *P*<0.0001; Figure 3). No significant interaction was observed by baseline LDL‐C (*P* for interaction=0.11).

**Figure 4:**
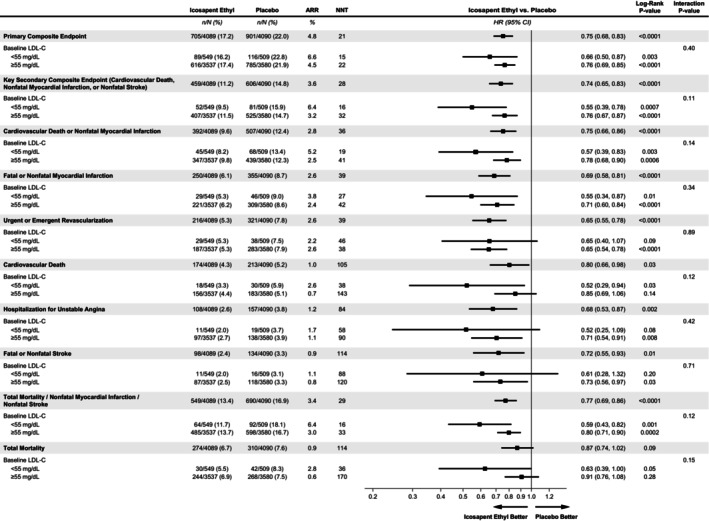
Forest plot of primary and secondary end points by baseline LDL‐C. Presented is the forest plot of the primary and secondary end points. Patients were stratified by baseline LDL‐C (<55 mg/dL versus ≥55 mg/dL). Patients were randomized to icosapent ethyl (2 g twice daily) or placebo. The primary composite end point included cardiovascular death, nonfatal myocardial infarction, nonfatal stroke, coronary revascularization, or unstable angina. A hazard ratio <1 indicates benefit with icosapent ethyl. ARR indicates absolute risk reduction; LDL‐C, low‐density lipoprotein cholesterol; and NNT, number needed to treat.

Similar findings were observed for the other secondary end points. Every cardiovascular end point examined, including composite end points, and the individual ones—MI, stroke, urgent or emergent revascularization, cardiovascular death, and hospitalization for unstable angina (with the sole exception of total mortality rate)—were reduced with icosapent ethyl compared with placebo in the overall group, and there was no significant interaction between treatment and baseline LDL‐C (>55 mg/dL versus <55 mg/dL; Figure 4). All‐cause death trended toward improvement with icosapent ethyl compared with placebo in the overall group (HR, 0.87 [95% CI, 0.74–1.02]; ARR, 0.9%; *P*=0.09), with no significant interaction with baseline LDL‐C (*P* for interaction=0.15).

### Safety End Points

Safety end points by baseline LDL‐C group were consistent with the overall study group. Similar rates of severe TEAEs (19.7% versus 20.0%; *P*=0.78) and drug‐related TEAE (12.6% versus 12.2%; *P*=0.61) were observed in the icosapent ethyl and placebo groups, respectively, without significant difference by baseline LDL‐C (Table S2). The icosapent ethyl group had an increased rate of atrial fibrillation/flutter (5.8% versus 4.5%; *P*=0.008), and a trend toward an increased rate of serious adverse bleeding (2.7% versus 2.1%; *P*=0.06), which were similar by baseline LDL‐C group (Table S2).

### Sensitivity Analyses

Among patients with a history of cardiovascular disease, icosapent ethyl compared with placebo reduced rates of the primary outcome (Figure S1) among those with LDL‐C <55 mg/dL (HR, 0.56 [95% CI, 0.41–0.77]; ARR, 11.6%; *P*=0.0002) and those with LDL‐C ≥55 mg/dL (HR, 0.75 [95% CI, 0.67–0.85]; ARR, 5.4%; *P*<0.0001). No significant interaction was observed between treatment and LDL‐C group (*P* for interaction=0.10).

In analyses of patients stratified by a higher LDL‐C threshold (70 mg/dL), findings were consistent with the primary analyses. Baseline characteristics by LDL‐C group are presented in Table S3. Among those with LDL‐C <70 mg/dL, icosapent ethyl reduced the rate of the primary composite end point (HR, 0.70; [95% CI, 0.60–0.83]; ARR, 5.6%; *P*<0.0001; Figure S2). In patients with LDL‐C ≥70 mg/dL, icosapent ethyl similarly reduced the rate of the primary composite end point (HR, 0.79 [95% CI, 0.69–0.89]; ARR, 4.2%; *P*=0.0002). No significant interaction was observed by baseline LDL‐C (*P* for interaction=0.29). These findings were consistent across the secondary end points (Figure S2).

No significant interaction was observed by LDL‐C spline and treatment group in the primary composite outcome (*P* for interaction=0.59; Figure 2). Nor was there a significant interaction by spline for the key secondary composite outcome (*P* for interaction=0.43; Figure S3). Similarly, none of the 8 other secondary outcomes in the prespecified testing hierarchy had a significant interaction by spline (each had *P* for interaction ≥0.18; Figure S3). Likewise, when LDL‐C was analyzed as a linear variable, all interactions were nonsignificant for all 10 end points (each had *P* for interaction ≥0.12; Table S4).

## Discussion

Icosapent ethyl reduced the rate of cardiovascular outcomes in statin‐treated patients with elevated triglycerides irrespective of baseline LDL‐C. These results were consistent across various secondary cardiovascular outcomes and in multiple sensitivity analyses, including with a different baseline LDL‐C threshold and in patients with cardiovascular disease history.

Patients with well‐managed baseline LDL‐C, to levels less than 55 mg/dL, benefited similarly from icosapent ethyl therapy as those with LDL‐C above the target. Icosapent ethyl demonstrated a large 34% reduction in cardiovascular events in patients with baseline LDL‐C <55 mg/dL. Additionally, spline analyses suggested benefits of icosapent ethyl down to the lowest values of baseline LDL‐C (as low as 40 mg/dL). These findings support that icosapent ethyl can prevent many cardiovascular events in patients with elevated triglycerides who are at high cardiovascular event risk.

Most contemporary lipid therapies for cardiovascular event prevention have focused on LDL‐C control.^25^, ^26^, ^27^ However, the current study emphasizes that among patients with elevated triglycerides, icosapent ethyl can be an effective additional strategy to further improve cardiovascular outcomes, even among statin‐treated patients achieving excellent LDL‐C control. Prior evidence has shown consistency of icosapent ethyl benefits across statin types, further supporting the independent benefits from LDL‐C control.^28^ Our findings suggested a numerically higher risk reduction of the primary outcome in the lower LDL‐C group in comparison with the higher LDL‐C group (34% versus 24%), which could be a marker of improved adherence to both statins and icosapent ethyl. However, these findings are challenging to interpret, as the difference is not statistically significant, and thus should be considered exploratory. Further studies to evaluate if this signal is real are necessary.

Despite these compelling findings, and the demonstrated cost effectiveness of icosapent ethyl,^29^, ^30^ clinical use of icosapent ethyl remains discouragingly low, even in countries where it has been approved and widely available for several years.^31^ Concerted efforts to identify and decrease barriers to icosapent ethyl therapy are important for improving cardiovascular outcomes in eligible patients, including any barriers in cost‐related access.^32^ Strategies to initiate therapy among indicated patients at index admission for acute coronary syndrome are likely to be effective and warranted.^33^ Surveillance data have demonstrated that the prevalence of mild to moderately elevated triglyceride levels in the United States continues to remain high,^34^ and icosapent ethyl should be considered to reduce cardiovascular events in such patients who are at high cardiovascular risk.

Low adoption could also reflect current clinical uncertainty for whether very low LDL‐C itself lessens residual risk in this population and, if not, whether icosapent ethyl continues to lower risk across a range of cardiovascular outcomes at such low LDL‐C levels. Our findings indicate that icosapent ethyl has beneficial effects independent of the many known contributions of LDL‐C to atherosclerosis. Icosapent ethyl is known to improve triglyceride levels,^35^ with REDUCE‐IT demonstrating 20% reduction in triglycerides with therapy.^3^ While the cardiovascular benefits of icosapent ethyl therapy might be in some part secondary to triglyceride lowering, data from REDUCE‐IT strongly suggest that other mechanisms are likely significant contributors.^36^ In addition to triglyceride control, higher EPA levels can lead to cardiovascular benefit.^37^ Analyses of REDUCE‐IT showed no difference in cardiovascular event reduction with icosapent ethyl whether the on‐treatment triglyceride levels were above or below 150 mg/dL.^3^

Importantly, in addition to this finding within REDUCE‐IT, the STRENGTH (Long‐Term Outcomes Study to Assess Statin Residual Risk With Epanova in High Cardiovascular Risk Patients With Hypertriglyceridemia) trial, which tested mixed EPA plus docosahexaenoic acid (omega‐3), and the PROMINENT (Pemafibrate to Reduce Cardiovascular Outcomes by Reducing Triglycerides in Patients With Diabetes) trial, which tested pemafibrate, showed lack of improvement in cardiovascular outcomes with triglyceride lowering among patients with hypertriglyceridemia.^38^, ^39^ Similar findings were observed among trials assessing niacin for triglyceride lowering.^40^, ^41^, ^42^ Thus, non–triglyceride‐related mechanisms are likely important to explain the cardiovascular benefit of icosapent ethyl in REDUCE‐IT.^43^, ^44^, ^45^ Additionally, irrespective of the biomarker pathway, imaging findings in the EVAPORATE (Effect of Vascepa on Improving Coronary Atherosclerosis in People With High Triglycerides Taking Statin Therapy) trial showed that icosapent ethyl led to a reduction in low‐attenuation plaque on coronary computed tomography angiography.^46^ Coronary plaque regression has been associated with improvement in cardiovascular outcomes^47^ and may be the pathway leading to beneficial effects with icosapent ethyl.^48^

Similar to REDUCE‐IT, the JELIS (Japan EPA Lipid Intervention Study) trial showed benefits with EPA, with the latter study including patients with hyperlipidemia and increased inflammatory state.^49^ In subgroup analyses, JELIS demonstrated no significant heterogeneity in treatment effect when stratifying patients by a LDL‐C threshold of ≈180 mg/dL (4.7 mmol/L).^49^ Our study adds evidence for the beneficial effects of EPA among those with much lower LDL‐C levels. Similarly, the RESPECT‐EPA (Randomized Trial for Evaluation in Secondary Prevention Efficacy of Combination Therapy–Statin and Eicosapentaenoic Acid) trial suggested improved cardiovascular outcomes with icosapent ethyl among patients with chronic coronary artery disease.^50^ RESPECT‐EPA subgroup analyses showed consistent benefit with EPA when patients were stratified by baseline LDL‐C of 70 mg/dL.^50^ REDUCE‐IT, JELIS, and RESPECT‐EPA, when compared with STRENGTH, used higher‐dose EPA formulations,^3^, ^39^, ^49^ with evidence indicating a relationship between EPA and cardiovascular outcome reduction.^37^, ^51^, ^52^

Although expected, it is reassuring to note that TEAE rates were similar by baseline LDL‐C group as for the overall trial, without significant differences in severe TEAEs or drug‐related TEAEs. It is also important to note that increased rates of atrial fibrillation or flutter, and a trend toward increased rates of serious adverse event bleeding were observed with icosapent ethyl in both LDL‐C strata, though absolute risk differences for serious adverse event bleeding were low in both cases. Overall, these findings continue to suggest the safety of icosapent ethyl as seen in prior REDUCE‐IT publications, although physicians will need to consider possible adverse effects when prescribing therapy.

We acknowledge the limitations of the analyses presented. First, this study was a post hoc analysis. Second, randomization was not stratified by baseline LDL‐C, though reassuringly we found that baseline characteristics were similar among the different baseline LDL‐C study groups. Third, REDUCE‐IT patients had very low use of ezetimibe and PCSK9 inhibitors. Fourth, REDUCE‐IT only enrolled patients with well‐controlled or mild to moderate elevations of LDL‐C (LDL‐C ≤100 mg/dL).

In conclusion, icosapent ethyl reduced the rate of cardiovascular events among statin‐treated patients with high cardiovascular risk and elevated triglycerides, irrespective of baseline LDL‐C. The large and statistically significant reduction in cardiovascular end points with icosapent ethyl in patients with LDL‐C <55 mg/dL suggests that this agent should also be considered for cardiovascular event prevention in statin‐treated patients with elevated cardiovascular risk despite well‐controlled LDL‐C.

## Sources of Funding

REDUCE‐IT was funded by Amarin, as were these analyses. Dr. Aggarwal receives research training support from a National Heart, Lung, and Blood Institute training grant (5T32HL007604).

## Disclosures

Dr Aggarwal is involved in research funded by the Bristol Myers Squibb–Pfizer alliance, Novartis, Lexicon, and Amarin, and has previously served as a consultant for Lexicon. Dr Bhatt has received research funding from Amarin paid to Brigham and Women's Hospital and the Icahn School of Medicine for his role as Chair of REDUCE‐IT (Reduction of Cardiovascular Events with Icosapent Ethyl–Intervention Trial) and discloses the following relationships: Advisory Board: Angiowave, Bayer, Boehringer Ingelheim, CellProthera, Cereno Scientific, Elsevier Practice Update Cardiology, E‐Star Biotech, High Enroll, Janssen, Level Ex, McKinsey, Medscape Cardiology, Merck, MyoKardia, NirvaMed, Novo Nordisk, PhaseBio, PLx Pharma, Stasys; Tourmaline Bio; Board of Directors: American Heart Association New York City, Angiowave (stock options), Bristol Myers Squibb (stock), DRS.LINQ (stock options), High Enroll (stock); Consultant: Broadview Ventures, GlaxoSmithKline, Hims, SFJ, Youngene; Data Monitoring Committees: Acesion Pharma, Assistance Publique‐Hôpitaux de Paris, Baim Institute for Clinical Research (formerly Harvard Clinical Research Institute, for the PORTICO trial, funded by St. Jude Medical, now Abbott), Boston Scientific (Chair, PEITHO trial), Cleveland Clinic, Contego Medical (Chair, PERFORMANCE 2), Duke Clinical Research Institute, Mayo Clinic, Mount Sinai School of Medicine (for the ENVISAGE trial, funded by Daiichi Sankyo; for the ABILITY‐DM trial, funded by Concept Medical; for ALLAY‐HF, funded by Alleviant Medical), Novartis, Population Health Research Institute; Rutgers University (for the National Institutes of Health–funded MINT trial); Honoraria: American College of Cardiology (Senior Associate Editor, Clinical Trials and News, ACC.org; Chair, ACC Accreditation Oversight Committee), Arnold and Porter law firm (work related to Sanofi/Bristol‐Myers Squibb clopidogrel litigation), Baim Institute for Clinical Research (formerly Harvard Clinical Research Institute; RE‐DUAL PCI clinical trial steering committee funded by Boehringer Ingelheim; AEGIS‐II executive committee funded by CSL Behring), Belvoir Publications (Editor‐in‐Chief, *Harvard Heart Letter*), Canadian Medical and Surgical Knowledge Translation Research Group (clinical trial steering committees), CSL Behring (American Heart Association lecture), Cowen and Company, Duke Clinical Research Institute (clinical trial steering committees, including for the PRONOUNCE trial, funded by Ferring Pharmaceuticals), HMP Global (Editor‐in‐Chief, *Journal of Invasive Cardiology*), *Journal of the American College of Cardiology* (Guest Editor; Associate Editor), K2P (Co‐Chair, interdisciplinary curriculum), Level Ex, Medtelligence/ReachMD (CME steering committees), MJH Life Sciences, Oakstone CME (Course Director, Comprehensive Review of Interventional Cardiology), Piper Sandler, Population Health Research Institute (for the COMPASS operations committee, publications committee, steering committee, and USA national coleader, funded by Bayer), WebMD (CME steering committees), Wiley (steering committee); Other: *Clinical Cardiology* (Deputy Editor); Patent: Sotagliflozin (named on a patent for sotagliflozin assigned to Brigham and Women's Hospital who assigned to Lexicon; neither I nor Brigham and Women's Hospital receive any income from this patent); Research Funding: Abbott, Acesion Pharma, Afimmune, Aker Biomarine, Alnylam, Amarin, Amgen, AstraZeneca, Bayer, Beren, Boehringer Ingelheim, Boston Scientific, Bristol‐Myers Squibb, Cardax, CellProthera, Cereno Scientific, Chiesi, CinCor, Cleerly, CSL Behring, Eisai, Ethicon, Faraday Pharmaceuticals, Ferring Pharmaceuticals, Forest Laboratories, Fractyl, Garmin, HLS Therapeutics, Idorsia, Ironwood, Ischemix, Janssen, Javelin, Lexicon, Lilly, Medtronic, Merck, Moderna, MyoKardia, NirvaMed, Novartis, Novo Nordisk, Otsuka, Owkin, Pfizer, PhaseBio, PLx Pharma, Recardio, Regeneron, Reid Hoffman Foundation, Roche, Sanofi, Stasys, Synaptic, The Medicines Company, Youngene, 89Bio; Royalties: Elsevier (Editor, *Braunwald's Heart Disease*); Site Coinvestigator: Abbott, Biotronik, Boston Scientific, CSI, Endotronix, St. Jude Medical (now Abbott), Philips, SpectraWAVE, Svelte, Vascular Solutions; Trustee: American College of Cardiology; Unfunded Research: FlowCo. Dr Steg has received grants, personal fees, and nonfinancial support from Sanofi; has received grants and personal fees from Amarin, Servier, and Bayer; and has received personal fees from Amgen, AstraZeneca, Bristol Myers Squibb, Boehringer Ingelheim, Idorsia, Pfizer, and Novartis. Dr Miller has served as a scientific advisor for Amarin Corp, 89bio, and Ionis. Dr Brinton has received speaker fees from Amarin, Amgen, Amryt, and Esperion; and has received consulting fees from 89Bio, Amarin, Amgen, Amryt, DelCor, Esperion, Immunovant, Ionis, Merck, NovoNordisk, and Pfizer. Dr Dunbar is an employee and stockholder of Amarin Pharma, Inc. Dr Ketchum is an employee and stockholder of Amarin Pharma, Inc. Dr Tardif has received research grants from Amarin, Ceapro, DalCor Pharmaceuticals, Esperion, Ionis, Merck, Novartis, and Pfizer; honoraria from DalCor Pharmaceticals, HLS Pharmaceuticals, Pendopharm, and Pfizer; minor equity interest from DalCor Pharmaceuticals; and authorship of patents on pharmacogenomics‐guided CETP inhibition and use of colchicine after myocardial infarction (Dr Tardif has waived his rights in the colchicine patent and does not stand to gain financially). Prof. Martens has no disclosures regarding this publication, but received research grants from Amarin, Amgen, Novartis, Novo Nordisk, and Sanofi paid to the involved institutions. Dr Ballantyne has received grant and research support (all significant and paid to the institution) from Abbott Diagnostic, Akcea, Amgen, Arrowhead, Esperion, Ionis, Merck, Novartis, Novo Nordisk, Regeneron, Roche Diagnostic, the National Institutes of Health, the American Heart Association, and the American Diabetes Association; is a consultant (modest except as noted) for 89Bio, Abbott Diagnostics, Alnylam Pharmaceuticals, Althera, Amarin, Amgen, Arrowhead, AstraZeneca, Denka Seiken (significant), Esperion, Genentech, Gilead, Illumina, Ionis, Matinas BioPharma, Merck, New Amsterdam (significant), Novartis, Novo Nordisk, Pfizer, Regeneron, and Roche Diagnostic. Dr Szarek reports serving as a consultant or research support (or both) from CiVi, Resverlogix, Lexicon, Baxter, Esperion, Amarin, NewAmsterdam, Silence, Tourmaline, Sanofi, and Regeneron Pharmaceuticals. Dr Mason has received research funding or consulting fees from Amarin, Lexicon, Esperion, and HLS Therapeutics.

## Supporting information

Data S1
Tables S1–S4
Figures S1–S3
